# Radical Cure of Artificial Anus

**Published:** 1871-02

**Authors:** 


### RADICAL CURE OF ARTIFICIAL ANUS.

Translated from the Gazette Hebdomadaire, by F. W. DRAPER, M. D., Boston.

M. Goyard recommends a new method of operation for the radical cure of artificial anus. "The real indication to be fulfilled," he says, "is to prevent the escape of the intestinal contents between the lips of the opening." For this purpose a suture is passed near the margin of the orifice, so that it is placed deeply and draws together tightly the hardened and callous cellular tissue which forms the wall of the canal. The suture, which should be strong and smooth, is passed deeply from side to side, just below the lower extremity of the orifice; then subcutaneously a little distance, then back again to the other side, and so on until the opening of the anus is all involved in this series of transverse stitches; a second suture passed in the opposite direction, the same points of entrance and exit in the skin being observed, completes the circuit and enables the operator to readily appose and retain the walls of the fistula. The needle used is one of moderate curve, with the eye near the point. After the sutures are passed, the surface of the fistulous tract is thoroughly refreshed and its external edge is drawn together by an ordinary interrupted suture.

The writer concludes very candidly: "The lesion is thus reduced to a simple wound, and we ought to expect union by first intention, if the general condition of the patient is good. Concerning the results of this method only conjecture is at present possible, since the confirmation of experience, without which all theories are good for nothing, is as yet absolutely wanting. One argument in its favor may, however, be offered; the patient is not obliged to undergo an operation, properly so called, and does not suffer any loss of tissue."—*Boston Medical and Surgical Journal.*

